# Demonstrating the value of cancer biomarkers at the population level

**DOI:** 10.1007/s10198-022-01474-6

**Published:** 2022-05-13

**Authors:** Afschin Gandjour

**Affiliations:** Frankfurt School of Finance and Management, Adickesallee 32-34, 60322 Frankfurt am Main, Germany

**Keywords:** Cancer, Biomarker, Value, I10, O33

Luís and Seo present a study that aims at “assess[ing] how the introduction of biomarker tests guiding cancer therapy have affected the premature mortality and survival of cancer patients in Norway [1].” To this end, the authors “make use of the fact that they [biomarkers—author's note] were introduced for different cancers at different points in time.” The authors present two different models. Model 1 starts from the assumption that “[b]iomarker-guided therapies can (…) be targeted to particular types of patients who would otherwise not have an effective treatment available”. Model 2 is based on the assumption that biomarker-guided therapies “can also (…) avoid[…] adverse reactions in potential nonresponders who ultimately do not consume these guided drugs.” The “analysis is based on patient-level data from the Cancer Registry of Norway and on drug sales data”.

I found the research question on the impact of biomarker tests on premature mortality/survival at the population-level quite intriguing. However, as the analysis derives the value of biomarker tests only from analyzing the uptake of biomarker tests and biomarker-targeted therapies over time, it is at best incomplete. Let me outline the two situations in which biomarker tests are able to provide value. In the first, the therapy has no or a negative impact on survival in the biomarker-negative population. When applied to the all-comer population (i.e., regardless of biomarker status), the therapy does not provide a clinically significant survival benefit and would not be approved. Therefore, the value of a biomarker is determined by comparing—in terms of survival—the uptake of a specific biomarker with no uptake (i.e., the prior situation). This value component of biomarkers appears to be appropriately addressed by Model 1 (with the usual caveats associated with these types of studies).

In the second situation, the therapy, if applied to the all-comer population, yields a clinically significant survival benefit, but the effect in the biomarker-negative population is smaller than in the biomarker-positive population. In this situation, the manufacturer may seek approval in the biomarker-positive population only for commercial reasons. If so, the value of a biomarker is ambiguous: on one hand, the biomarker reduces the survival benefit, because responders in the biomarker-negative population do not receive the therapy.^1^ On the other hand, the biomarker increases the survival benefit, because patients in the biomarker-negative population are spared from potential drug toxicities causing death (i.e., grade 5 adverse events). However, to determine the net medical effect (benefit minus harm) requires data on a counterfactual, where the drug is provided in the all-comer population. Yet, this information can only be provided by clinical trials that have tested the therapy in the all-comer population including those patients who are biomarker negative. Hence, this information cannot be retrieved from real-world data, because the latter do not contain information on the effects and adverse events in the non-approved, biomarker-negative population. Neither do they include information on the size of the biomarker-negative population, which is needed to calculate the net survival impact at a population level.^2^ Therefore, this value of biomarkers has not been appropriately assessed by Model 2. While Model 2 aims at comparing targeted and non-targeted therapies, it does not compare the same drug in the same patient population, which is the necessary condition for a valid comparison. The only theoretical exception would be a class-effect of all new biomarker-targeted therapies in terms of survival benefits and adverse events, which, of course, does not align with the real-world situation. Hence, the study does not allow determining the full impact of biomarker tests on survival.

Finally, it needs to be emphasized that the net medical effect (benefit minus harm) of introducing a biomarker is negative if patients in the biomarker-negative population forgo therapy with a small but not clinically relevant benefit. In this case, the value provided by a biomarker entirely lies in cost savings to the healthcare system.^3^ Hence, it would be a mistake to believe—and reading the publication conveys this impression—that a positive value of biomarkers hinges upon a positive impact on survival.

## Footnotes

1 The authors are aware of this fact by stating: “Those patients could be deprived of potentially beneficial drugs that target their specific type of cancer better or have an incremental improvement in health relative to earlier drugs.”.

2 Of note, the presence of off-label use in the biomarker-negative population would still require information on the size of the biomarker-negative population without off-label use and the foregone survival benefit in this subpopulation.

3 The study mentions the goal of “limit[ing] the expenditure ineffective therapies” only in passing and does not determine savings (notwithstanding the question around a valid counterfactual).
